# Rules for body fat interventions based on an operating point mechanism

**DOI:** 10.1016/j.isci.2023.106047

**Published:** 2023-01-25

**Authors:** Alon Bar, Omer Karin, Avi Mayo, Danny Ben-Zvi, Uri Alon

**Affiliations:** 1 Department Molecular Cell Biology, Weizmann Institute, Rehovot, Israel; 2 Department of Mathematics, Imperial College London, London, SW7 2AZ, UK; 3 Department of Developmental Biology and Cancer Research, Institute for Medical Research Israel Canada, Hebrew University-Hadassah Medical School, Jerusalem, Israel

**Keywords:** Biological sciences, Physiology, Human metabolism, Endocrinology, Mathematical biosciences

## Abstract

Interventions to reduce fat are important for human health. However, they can have opposing effects such as exercise that decreases fat but increases food intake, or coherent effects such as leptin resistance which raises both. Furthermore, some interventions show an overshoot in food intake, such as recovery from a diet, whereas others do not. To explain these properties we present a graphical framework called the operating point model, based on leptin control of feeding behavior. Steady-state fat and food intake is given by the intersection of two experimental curves – steady-state fat at a given food intake and *ad libitum* food intake at a given fat level. Depending on which curve an intervention shifts, it has opposing or coherent effects with or without overshoot, in excellent agreement with rodent data. The model also explains the quadratic relation between leptin and fat in humans. These concepts may guide the understanding of fat regulation disorders.

## Highlights

• A mathematical model for regulation of fat mass in rodents and humans
• The model consists of two measurable curves: steady-state fat and *ad libitum* food intake
• The model predicts patterns of fat mass change and food intake after interventions
• Anti-starvation mechanism maximises intake at low fat level

## Graphical abstract

**Figure:**
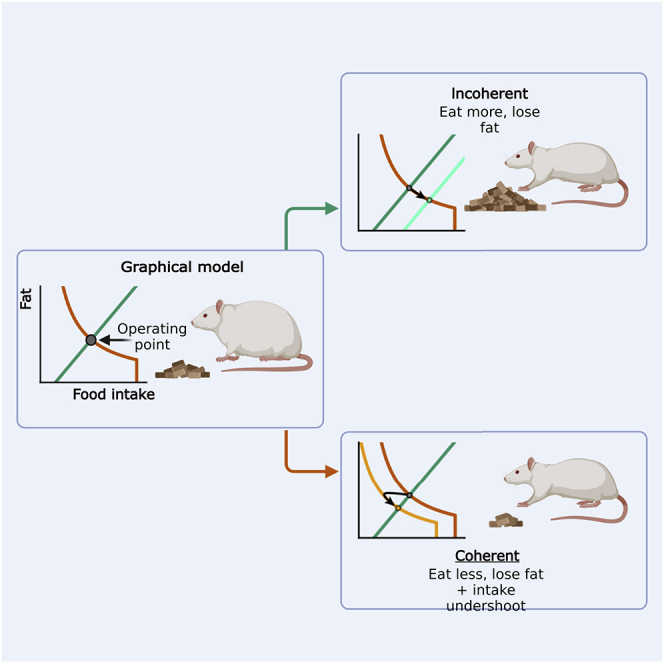

## Introduction

Obesity, an excessive accumulation of fat that can impair health, is a global epidemic whose prevalence has nearly tripled over the past 50 years.^1^ Obesity is associated with increased risk of type 2 diabetes, cardiovascular diseases, respiratory disorders, infertility, and some forms of cancers.^2^ Its etiology includes interactions among genes, hormones, the nervous system, and the environment.^3^

Understanding the regulation of fat is therefore imperative, and interventions or conditions that affect fat mass have been extensively studied, especially in rodents. The interventions show a complex pattern. Some cause opposing effects on food intake and fat mass, as in like exercise and hyperthyroidism which decrease fat mass despite an increase in food intake.^4^^,^^5^^,^^6^^,^^7^^,^^8^^,^^9^^,^^10^ Other interventions show coherent effects in which fat and food intake change in the same direction, such as inducing leptin resistance which makes both fat and food intake rise.^11^^,^^12^^,^^13^^,^^14^^,^^15^ Principles are needed that can explain when the effects on fat and food intake are coherent and when they are opposing.

There is also complexity in the temporal profiles of food intake after an intervention. Some interventions, like administration of leptin antagonists, show an overshoot in food intake that lasts a few days before settling down to a new steady-state.^12^^,^^14^^,^^16^ Other interventions, like exercise, show no overshoot or undershoot.^8^ We lack principles to understand when an overshoot occurs, and what factors affect the dynamics of feeding after an intervention.

On the molecular level, fat mass is controlled in multiple ways.^17^^,^^18^^,^^19^ Hormones and neuropeptides affect feeding behavior and metabolic rate on several timescales, from the scale of minutes to the scale of months, where metabolic rate can show long-term changes such as adaptive thermogenesis.^20^^,^^21^^,^^22^ One feedback regulator of appetite that acts within hours to days is the hormone leptin.^23^ Leptin is produced by adipocytes, and its serum concentration rises approximately quadratically with fat mass.^24^ Leptin thus is a signal that circulates in the blood to represent the body’s available energy stores. It interacts with receptors in the brain to suppress food intake and to promote growth, energy expenditure and glycemic control.^19^

Existing concepts^25^^,^^26^ and mathematical models^27^^,^^28^ do not explicitly provide rules that connect the molecular level circuits to the systemic effects of interventions mentioned above. It would therefore be important to understand the observed effects of interventions in terms of molecular mechanisms within a conceptual framework that can guide new experiments.

Here we present a simple graphical and mathematical model that provides rules for interventions that affect fat mass. We test these predictions against a wide range of previous experiments in rodents. The model is based on the properties of two experimentally measurable curves — steady-state fat at a given food intake and *ad libitum* food intake at a given fat level. The intersection of the two curves provides the steady-state fat and food intake of a given individual, which we call the operating point. Each intervention shifts one or the other curve. Depending on which curve is shifted, the model provides rules for whether the effect on food intake and fat is opposing or coherent, and whether an overshoot in food intake will occur. The model explains a wide range of intervention effects and provides the quadratic relation between leptin and fat in rodents and humans. It identifies the key physiological parameters that underlie differences between individual operating points and provides a systematic framework to understand strategies to regulate body fat.

## Results

### An operating point model for food intake and fat mass

**Figure 1:**
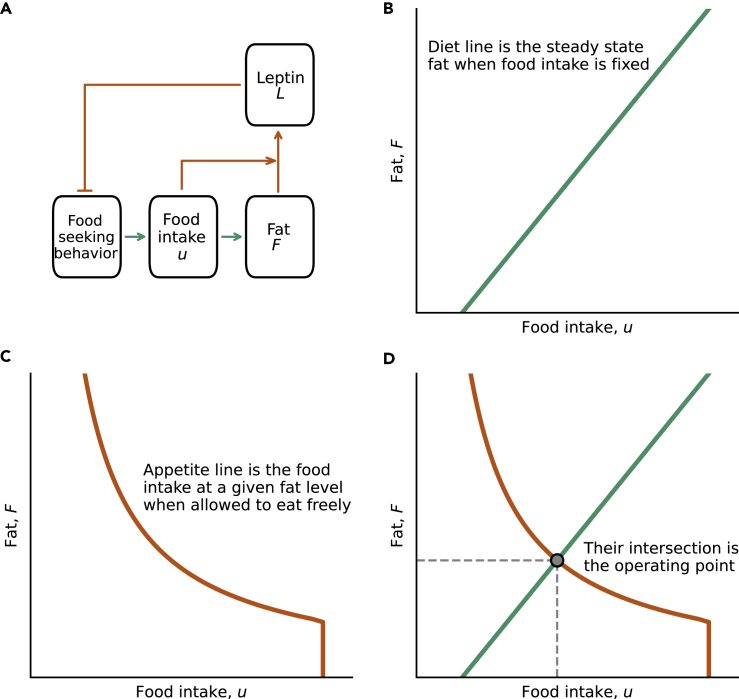
The operating point lies at the intersection of the diet and appetite lines (A) The leptin system provides a feedback loop that controls fat by regulating food intake. The circuit contains two arms that can be analyzed independently. (B) The first arm is how food intake leads to more fat. An experimentally accessible line is steady-state fat at a given controlled food intake. This is the diet line. (C) The second arm is the food intake over one day at a given level of fat, when the organism is allowed to eat as much and as often as it wants. (D) The crossing point of these lines is the steady state of the system which we call, following engineering tradition, the operating point.

We propose a conceptual model of fat regulation based on physiological feedback between fat and feeding behavior. The model concerns fat mass changes over the scale of days to weeks, rather than the control of feeding on the faster timescale of meals. A working model for this feedback is shown in Figure 1A, where leptin is secreted by fat mass to reduce food intake. The model is agnostic, however, to the precise molecular nature of the feedback loop, which can include additional regulators.

The feedback loop operates in two arms, which we first analyze separately. In the first arm, food intake leads to more fat. This arm can be quantitated by an experimentally accessible curve: steady-state fat at a given controlled food intake. This steady-state might take days to weeks to reach. We call this curve the diet line (Figure 1B). It is a rising curve because the higher the controlled food intake, the higher the steady state fat. The diet line includes the conversion rate of food to fat, and the loss of fat because of basal metabolic rate and energetic costs of activity.

The second arm of the feedback loop describes how fat controls appetite. The relevant experimental curve is the food intake over one day at a given level of fat, when the organism is allowed to eat *ad libitum*, that is, as much and as often as it wants. The units of food intake can be either grams or calories per day. We call this curve the appetite line (Figure 1C). The curve is a declining curve because of the feedback control where fat mass inhibits food intake.

The precise shapes of the curves in Figure 1 are based on a mathematical model which we describe below. But for most of our conclusions, it only matters that the diet line rises (more food intake means more fat) and the appetite line falls (more fat means less food intake).

Next, we draw these two curves on the *phase portrait*, a graph whose axes are food intake and fat mass. The two lines cross at a point, which we call, following engineering tradition, the *operating point* (Figure 1D). We choose not to call it a set point or settling point to avoid confusion with existing concepts, as described in the discussion. The operating point is the steady-state fat level that leads to a level of food intake that precisely supports it.

To demonstrate how these curves can be experimentally measured we analyzed data on the body composition of female rats recovering from controlled overfeeding or underfeeding from Harris et al.^29^ Normal weight rats were tube-fed 160%, 100% or 40% of control food intake for 20 days. Then, the rats returned to *ad libitum* feeding. Weight and food intake were recorded daily, and fat content was evaluated at several time points.

**Figure 2:**
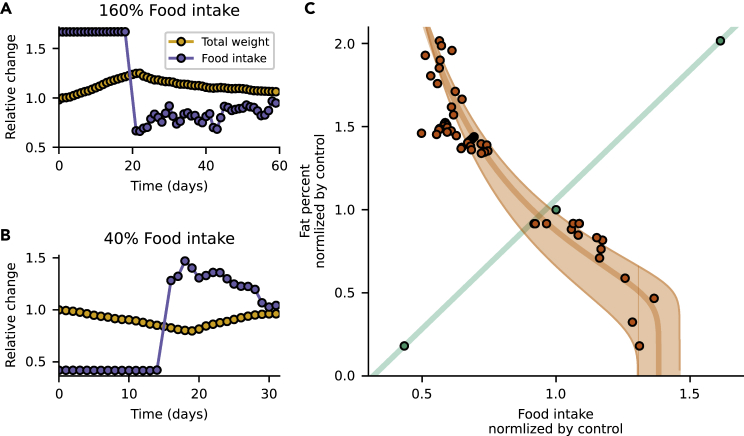
Controlled feeding experiments can measure the diet and appetite lines (A) Data adapted from Harris et al., 1986 on rats that were tube-fed 160% of normal food intake. The rats increased in weight. On recovery, an undershoot in food intake is observed. (B) Rats that were restricted to 40% of normal food intake reduced in weight. On recovery, an overshoot in food intake is observed. (C) Experimental phase portrait shows the diet (green dots) and appetite (orange dots) lines. Mathematical model is in full lines, with SD of parameters estimation.

Because rats gain weight as they grow, we normalized each experimental group by the control (Figures 2A and 2B). This also helped to correct for the adverse effect of tube feeding, evident in the 100% control group.

The controlled feeding part of the experiment determines the diet line, the steady-state fat at a given controlled food intake level. The experiments provide three points on the line. Each point is defined by the fat relative to control at the end of the 20 days of controlled feeding. The three points lie approximately on a straight line.

The recovery phase of the experiment enabled us to draw the appetite line. Each point on this line is defined by the relative fat and the relative daily *ad libitum* food intake (STAR Methods). The appetite line shows curvature at both ends (Figure 2C). The two curves cross at the operating point which defines normal fat and intake for these rats.

### Interventions that affect the diet line cause opposing effects on food intake and fat

This graphic approach based on the two curves predicts certain rules for how interventions should affect fat and food intake. We begin with interventions that affect the diet line. When the diet line moves, the operating point slides along the unchanged appetite line and shifts to a new position. Because the appetite line has a downwards slope, these alterations have opposing effects on food and fat. Fat rises and food intake drops, or vice versa.

**Figure 3:**
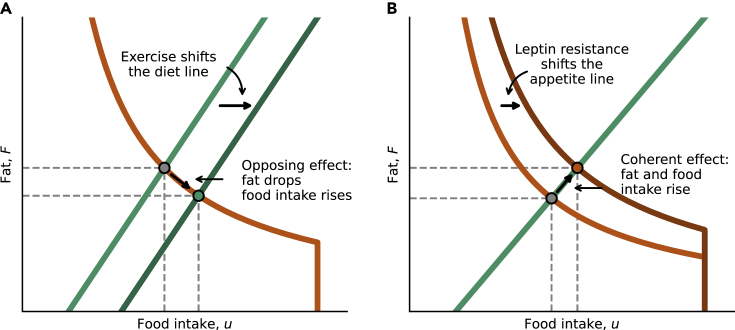
The two lines allow analysis of the effect of alterations on the operating point (A) Alterations in energy balance, shift the diet line and lead to opposing effects on food intake and fat. (B) Alterations in leptin signaling shift the appetite line and lead to coherent effects on food intake and fat.

For example, a rise in energy expenditure means that a given amount of food intake supports a lower steady-state fat mass. This shifts the diet line to the right. The operating point moves to a new position, where fat drops but food intake rises (Figure 3A). Fat and intake change in opposite directions.

To test this prediction, we analyzed the literature on intervention experiments in rodents that changed energy expenditure, keeping food composition constant. We tested exercise, hyperthyroidism and exposure to cold temperature, all of which raised energy expenditure. These conditions caused an increase in food intake and a decrease in fat mass (Table 1), as predicted.

**Table 1:** Experiments that shift the diet line show opposing effects on fat percentage and food intake in grams

Intervention	Fat/food intake	Reference
Exercise	Opposing: fat-/food+	Afonso and Eikelboom, Mayer et al., Mazzeo and Horvath, Mueller et al.^4^^,^^6^^,^^8^^,^^9^
Hyperthyroidism	Opposing: fat-/food+	Ishii et al., Luo and MacLean, Syed et al., Iglesias et al.^5^^,^^7^^,^^10^^,^^30^
Hypothyroidism	Opposing: fat+/food-	Iossa et al.^31^
Room to cold temperature	Opposing: fat-/food+	Bing et al., Cottle and Carlson, Hamilton and Brobeck, Leung and Horwitz, Zhang et al.^32^^,^^33^^,^^34^^,^^35^^,^^36^
Cold to room temperature	Opposing: fat+/food-	Cottle and Carlson^33^

Similarly, conditions that lowered energy expenditure, such as a return from prolonged cold temperature to room temperature, caused opposing effects in which fat increased but food intake decreased. We conclude that interventions that affect energy balance are correctly predicted to have opposing effects on intake and fat.

### Alterations in the appetite line lead to coherent effects on food intake and fat

In contrast to the opposing effects of alterations in the diet line, changes in the appetite line are predicted to show coherent changes in steady-state food intake and fat, in which both rise together or fall together. This is because shifting the appetite line moves the operating point along the unchanged diet line which is a rising curve. Both food intake and fat therefore shift in the same direction (Figure 3B) under such interventions.

We tested this prediction using rodent experiments that affect primarily the appetite line. These experiments included induced leptin resistance, leptin and leptin antagonist treatments, gastric bypass and satiety-inducing dietary fibers. All of these interventions showed the predicted coherent changes in food intake and fat (Table 2). For example, leptin treatment and dietary fibers reduced both intake and fat, and leptin antagonists increased both.

**Table 2:** Experiments that shift the appetite line show coherent effects on fat percentage and food intake in grams

Intervention	Fat/food intake	Reference
Leptin pathway inhibition	Coherent: fat+/food+	Della-Zuana et al., Fox et al., Guo et al.,Rushing et al., Waldbillig et al., Mack et al., Guo et al., Rushing et al., Waldbillig et al.^11^^,^^12^^,^^13^^,^^14^^,^^15^^,^^37^^,^^38^^,^^39^^,^^40^
Leptin pathway activation	Coherent: fat-/food-	Zhang et al., Dakin et al., Ikeda et al., Mack et al., Muta et al., Roth et al.^16^^,^^41^^,^^42^^,^^43^^,^^44^^,^^45^
Satiety inducing nutrients	Coherent: fat-/food-	Adam et al., 2014^46^; Fantini et al., 2009^47^; Zhou et al., 2011^48^
Gastric Bypass	Coherent: fat-/food-	Hao et al.^49^
Diet Variety	Coherent: fat+/food+	Louis-Sylvestre et al., Rogers and Blundell^50^^,^^51^

An additional intervention that affected appetite was increased variety of food, keeping caloric density constant. Generally, increased dietary variety causes animals become hyperphagic and increase body weight, even when dietary composition is controlled for by varying flavors and/or texture of the foods.^52^ These experiments show a coherent change in which both fat and food intake increase with variety. We conclude that interventions that affect the appetite curve are correctly predicted to affect intake and fat in the same direction.

### Eating overshoot occurs after perturbations that move away from the appetite line

So far we discussed rules about the steady-state effects of interventions on fat and food intake. We now consider the dynamics associated with a perturbation, namely the transient changes that occur along the return to an operating point. To understand these dynamics, we consider the time after an intervention starts or ends. The organism begins away from its eventual operating point, and its fat and food intake change over time toward the eventual operating point.

To understand the dynamics, it is useful to note a separation of timescales: Food intake changes much more rapidly than fat levels. Whereas intake changes immediately on the first day, fat accumulates more slowly and can take a week or more to reach its new steady state. We can use this separation of timescales to understand the dynamics.

Suppose that following a perturbation, the organism is with fat level $F_{0}$ and intake level $u_{0}$. We will call this the initial condition. If the initial condition is not on the appetite line, food intake will move on the first day to the appetite line. This is because of the definition of the appetite line as *ad libitum* intake at a given level of fat.

**Figure 4:**
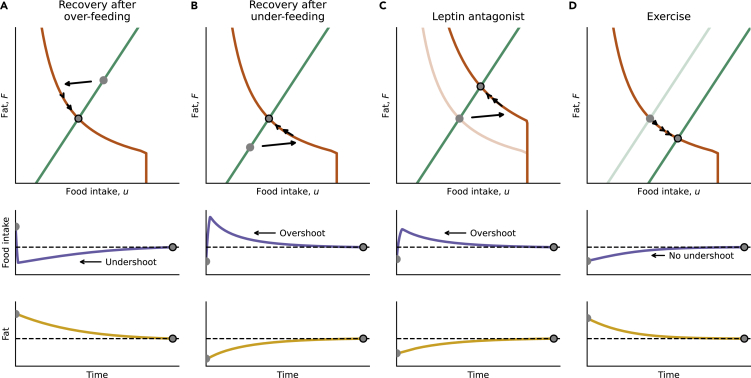
Food intake and fat dynamics show overshoots after certain perturbations and no overshoot after other perturbation (A) Undershoot of food intake during recovery from overfeeding: *ad libitum* intake drops to the appetite line, then slowly increases as fat is reduced. (B) Overshoot of food intake during recovery from under-feeding: intake rises to the appetite line, then slowly decreases as fat increases. (C) Overshoot of food intake during leptin antagonist treatment. Intake increases to the new appetite line, and then slowly decreases as fat increases. (D) No overshoot during adaptation to exercise. Exercise provides a low-fat, high-intake operating point. Food intake monotonically increases as fat slowly decreases.

Then, food intake and fat levels ‘crawl’ along the appetite line, as fat changes slightly every day and food intake is dictated by the fat level. Thus, an undershoot or overshoot in food intake is predicted when the initial condition is away from the appetite line. This occurs, for example, when *ad libitum* feeding begins after a period of controlled overfeeding (Figure 4A) or under-feeding (Figure 4B), or after an intervention that changes the appetite line, such treatment with leptin antagonist (Figure 4C).

For example, in recovery from overfeeding, fat starts at high levels. In the first days of *ad libitum* feeding, fat is still high which inhibits food intake. As fat levels slowly decrease over many days, intake follows fat on the appetite line (Figure 4A). This results in an undershoot in which food intake first drops and then rises. Similarly, an overshoot in intake occurs after controlled under-feeding. This is because fat starts low and disinhibits food intake. Food intake thus increases quickly, and then drops over many days as fat rises (Figure 4B).

Such undershoots and overshoots in intake are seen in the Harris et al. experiment.^29^ When the rats are allowed to eat *ad libitum* they demonstrate an intake overshoot after under-feeding and an intake undershoot after overfeeding (Figures 2A and 2B). We note that such hyperphagia after starvation can be modified also by the fat-free mass and other aspects of adaptive thermogenesis.^21^^,^^53^

In contrast, the model predicts no overshoot (that is monotonically rising or falling dynamics) when initial conditions are on the appetite line. As a result, food intake crawls along the appetite line without showing overshoots or undershoots. This is predicted to occur, for example, after an intervention that changes the diet line such as exercise, hyperthyroidism or changes in ambient temperature (Figure 4D)].

We tested these predictions using rodent experiments. We find an overshoot in most studies on leptin inhibition, and an undershoot after leptin activation or satiety inducing dietary fibers, as predicted for interventions that change the appetite line. An overshoot also occurred, as predicted, after a shift to high variety food with the same caloric density. No overshoot occurred after onset of interventions that affect the diet line, namely exercise, hyperthyroidism and cold temperature, as predicted (Table 3. See supplemental information for a detailed analysis.

**Table 3:** Experiments that shift the appetite or diet lines and show the predicted overshoot or undershoot in food intake

Intervention	Line affected	Overshoot	Reference
cold temperature	Diet	no	Cottle and Carlson, Hamilton and Brobeck^33^^,^^34^
Exercise	Diet	no	Mazzeo and Horvath^8^
Hyperthyroidism	Diet	no	Ishii et al., 2003^5^; Luo and MacLean, 2003^7^; Mazzeo et al., 1986^8^
Leptin pathway inhibition	Appetite	overshoot	Fox et al., 2007^12^; Zhang et al., 2007^16^; Rushing et al., 2001^14^
Leptin pathway activation	Appetite	undershoot	Fox et al., 2007^12^; Roth et al., 2006^45^
Satiety-inducing nutrients	Appetite	undershoot	Adam et al., Zhou et al., Fantini et al.^46^^,^^48^^,^^54^

To summarize the predicted rules, a change in a parameter that affects the diet line is predicted to show opposing effects on food intake and fat, as well as monotonic intake dynamics. A change in a parameter that affects the appetite line is predicted to show coherent effects on intake and fat and overshoot/undershoot food intake dynamics.

### Changes in food composition are best explained by considering intake in grams per day

So far, all interventions we have discussed kept food composition constant. We also considered interventions which changed food caloric density and composition. Such interventions may affect both the diet line and appetite line in principle. Because all of the interventions we discussed so far kept food composition constant, and hence it did not matter if food intake was measured in grams per day or calories per day. By analyzing changes in food composition, we sought to understand which of these two measures makes more sense in terms of the model.

Experimental changes in protein content showed coherent effects in fat mass and energy intake.^55^^,^^56^^,^^57^^,^^58^^,^^59^ For example, rodents consuming a high protein diet reduced their food intake in both grams and calories per day.^56^^,^^57^ They reached a lower body fat mass, and higher lean mass, compared to rodents who pair-fed the same amount of energy intake. Total body weight was similar. Energy intake and fat mass were reduced as a function of protein/lipid ratio in a carbohydrate-free diet, whereas intake in grams kept relatively constant.^58^

Protein generally has a greater satiating and thermic effects than carbohydrates.^60^^,^^61^^,^^62^ This suggests that protein composition and protein/lipid ratio primarily affect the appetite line, and that either grams or calories can be used.

Experiments on increased food fat content were more complicated to interpret. High fat content led to an increase in fat and to an increase in calories per day but a decrease in food grams per day.^13^^,^^16^^,^^63^ Rodents thus ate less food mass but more in terms of calories. To interpret this, we note that fat content strongly affects the diet line – each gram of high-fat food increases steady-state fat more than a gram of regular chow. The theory therefore predicts opposing effects. This suggests that to analyze such fat content interventions it makes sense to measure food intake in grams per day rather than calories per day.

We conclude that grams may be the most relevant measure of food intake for cases in which food composition varies. This choice makes no difference for the rules for the other interventions studied here.

### A minimal mathematical model for human leptin variation

So far we discussed experiments in rodents. The situation in humans is more complex because of physiological and cultural reasons. It is more difficult to obtain reliable data for the diet and appetite lines, because this requires controlled food intake over weeks in humans, with issues in reporting and adherence. Human physiology has more complex adaptive thermogenesis effects and differs from rodent physiology in many ways.^21^^,^^64^^,^^65^^,^^66^

The rules described above, however, seem to apply at least in some cases to humans. For example, hyperthyroidism is well known to cause fat reduction despite increased appetite^67^ Chronic exercise is accompanied by reduced fat and increased food intake.^68^^,^^69^ After a diet, a food intake overshoot is a common phenomenon,^70^ and so on.

**Figure 5:**
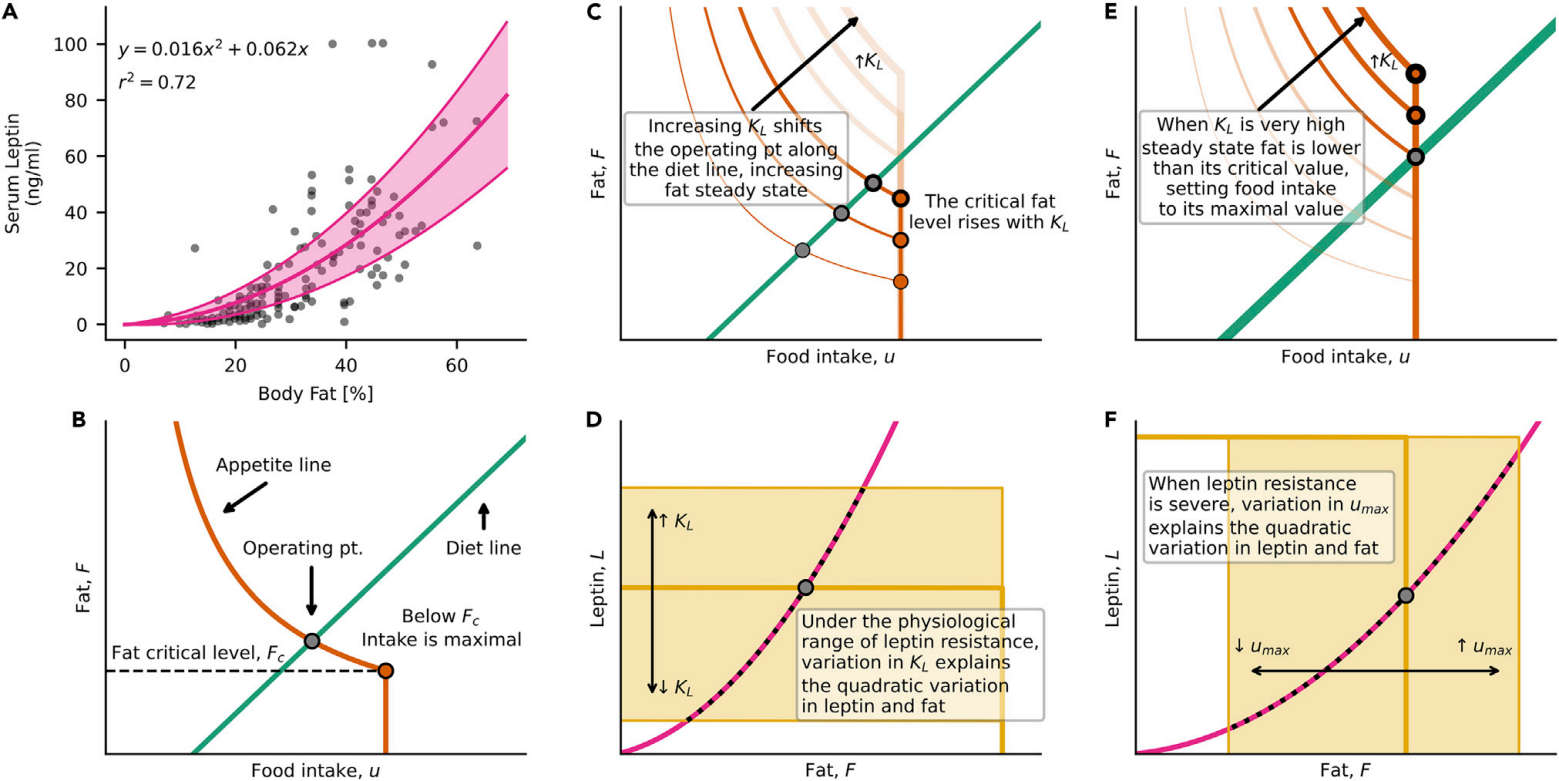
A minimal mathematical model for human leptin variation (A) Quadratic relation between leptin and fat in a human population, adapted from Considine et al., 1996.^24^ Best fit in full lines, with standard deviation of parameters estimation. (B) The diet and appetite curves form a minimal model of leptin action suggest that intake is inverse to fat, and locks to its maximal level when fat drops below a critical level. (C) Leptin resistance increases the steady state fat level, as long as fat is above the critical level $F_{c}$. The latter rises with leptin resistance. (D) In this regime, variation between individuals in leptin resistance accounts for the main difference in fat between individuals. (E) In severe leptin resistance, food intake hits a ceiling equal to the satiety food intake level $u_{\max}$. (F) In this regime, variations between individuals in the satiety food intake level $u_{\max}$ mainly account for differences in fat.

Humans offer an additional experimental fact however, that can add to our quantitative understanding of the control circuitry. This is based on extensive measurements of leptin *L* versus fat *F* in a population. This relation is approximately quadratic, L∼$F^{2}$^24^, with appreciable scatter around the quadratic curve (Figure 5A) (supplemental information). A quadratic relation is seen in several independent studies in different human populations.^12^^,^^24^^,^^71^^,^^72^^,^^73^

To understand the quadratic relation of fat and leptin requires a more mechanistic model, which provides specific shapes to the diet and appetite lines. So far the rules mentioned above did not require such precise shapes, and only required that the diet and appetite lines rise and fall respectively. We now use the human leptin data to develop a more specific model based on the leptin system.

The model describes the rates of change of fat and of leptin. The rate of fat gain from a food intake of u grams/day is $a_{F}u$. The parameter $a_{F}$ depends on food composition: $a_{F}$ Is higher for food rich in fat, for example, than in low fat food. Fat removal is because of the energy cost of fat itself, which is proportional to fat mass $\gamma_{F}F$, and to the energy cost of the rest of the body $\gamma_{E}$ , including basal metabolic rate and the cost of movement and exercise. The rate of change of fat is thus given by gain minus loss:

(Equation 1) $$\frac{dF}{dt}=a_{F}u-\gamma_{E}-\gamma_{F}F$$

At steady state, body fat does not change, namely, dF/dt=0. This provides a linear increase of fat with food intake. This is the diet line, in agreement with the linear shape of the rodent diet line in Figure 2.

(Equation 2) $$u=\frac{\gamma_{F}}{a_{F}}F+\frac{\gamma_{E}}{a_{F}}$$

Leptin, L, is secreted by fat F, in a way that increases with recent food intake.^74^^,^^75^^,^^76^^,^^77^ We thus model leptin production as a product of fat and intake: $a_{L}F$ u. Leptin is removed primarily by clearance in the kidney^78^ at rate $\gamma_{L}$. The difference between production and removal gives the rate of change of leptin:

(Equation 3) $$\frac{dL}{dt}=a_{L}Fu-\gamma_{L}L$$

The removal of leptin is rapid, with a half life of about 40 min$(\frac{\ln\left(2\right)}{\gamma_{L}}∼40\;\min$).^79^ Leptin dynamics are thus much faster than the fat dynamics which change over days. We can thus assume that leptin is at quasi steady-state, dL/dt=0:

(Equation 4) $$L_{qst}=\frac{a_{L}}{\gamma_{L}}Fu$$

Leading to a quadratic relation between leptin and fat (Substituting Equation 2 into Equation 4):

(Equation 5) $$L_{st}=\frac{a_{L}}{\gamma_{L}a_{F}}F\left(\gamma_{F}F+\gamma_{E}\right)$$

Note that if leptin production did not depend on recent food intake u, there would be a linear relation of fat and leptin, not a quadratic relation as observed.

Parameter values are listed in Table 4). Variation in the parameters of this equation can explain the variation around the quadratic relationship between leptin and fat [supplemental information]. For example, changes in food fat content that affect $a_{F}$ and changes in basal metabolic rate or exercise that affect $\gamma_{E}$ shift leptin at a given fat level.

**Table 4:** Parameter values used to model the operating point model

Parameter	Units	Default	Figure 3A (dark)	Figure 3B (dark)	Figure 4C	Figure 4D	Figure 5B	Figures 5C and 5E	Figure 5D	Figure 5F
$a_{F}$	$\frac{1}{40}minute^{-1}$	0.02	0.02	0.02	0.02	0.02	0.01	0.01	0.01	0.01
$\gamma_{F}$	$\frac{1}{40}minute^{-1}$	0.025	0.025	0.025	0.025	0.025	0.025	0.025	0.025	0.025
$\gamma_{E}$	$\frac{1}{40}minute^{-1}\cdot Mass_{fat}$	0.00625	0.0134	0.00625	0.00625	0.01875	0.00625	0.00625	0.00625	0.00625
$a_{L}$	$\frac{1}{40}minute^{-1}\frac{A.U.}{Mass_{fat}\cdot Mass_{intake}}$	1	1	1	1	1	1	1	1	1
$\gamma_{L}$	$\frac{1}{40}minute^{-1}$	1	1	1	1	1	1	1	1.5	1.5
$K_{L}$	A.U.	0.5	0.5	0.75	1	0.5	0.5	0.35–1.6	0.1–0.8	3.6
$u_{\max}$	$Mass_{intake}$	2	2	2	2	2	2	2	2	0.9–2.7

A.U. - Arbitrary units of leptin concertation. $Mass_{intake}$ – Arbitrary units of food intake mass. $Mass_{fat}$ – Arbitrary units of fat mass. Time measured in unites of leptin turnover, $\frac{1}{40}minute^{-1}$.

### Minimal model predicts parameters that affect human fat variation

This model can address the question of why people vary by about four-fold in fat levels. Which parameters might account for this variation? To address this requires completing the model by providing the link between leptin and food intake. To do so, we assume that leptin suppresses food intake according to a Hill function:

(Equation 6) $$u=\frac{u_{\max}}{1+{\left(\frac{L}{K_{L}}\right)}^{n}}$$

The halfway point is $K_{L}$, a parameter that rises in leptin resistance, and the steepness is determined by the Hill coefficient n. When there is no leptin, eating is at its maximal “satiety” value, $u_{\max}$. This maximal satiety is because of stomach distention, hormones like ghrelin and GLP-1, and other factors.^80^^,^^81^^,^^82^^,^^83^

This equation provides the appetite line (Plugging Equation 4 into Equation 6)

(Equation 7) $$F=\frac{\gamma_{L}K_{L}}{a_{L}}\frac{1}{u}\;{\left(\frac{u_{\max}}{u}-1\right)}^{\frac{1}{n}}$$

The theoretical line is similar to the appetite line from the rodent experiment of Figure 2, when n is large (n=7 is the best fit). This high n indicates a steep relationship between food intake and leptin levels. Physiologically, a steep relationship is supported by the finding that plasma leptin transport to the brain is saturable.^84^

Because steepness is high, it makes sense to simplify the formula by considering the limit of infinite steepness, n→∞. The appetite line has a simple mathematical form that has curvature at both ends, similar to the rodent appetite line of Figure 2. Food intake drops inversely with fat, but when fat reaches a lower limit $F_{c}$, food intake becomes locked at its maximum possible value $u_{\max}$(Figure 5B).

(Equation 8) $$u=\left\{ \begin{array}{c} \frac{F_{c}}{F}u_{\max},F>F_{c}\\ u_{\max},F\leq F_{c} \end{array}\right.,F_{c}=\frac{K_{L}\gamma_{L}}{a_{L}u_{\max}}$$

The maxing out of food intake causes an abrupt drop in the appetite line below the critical fat level $F_{c}$ (Figure 5B). This provides a strong defense against starvation. When body fat drops below a critical level, food intake is set to maximal, raising fat at the highest possible rate when food becomes available.

The critical fat level is proportional to $K_{L}$ (Figure 5C), the leptin halfway effect point, and therefore rises with leptin resistance. Leptin-resistant organisms are predicted therefore to go to maximal intake more readily (at a higher critical fat level) than non-resistant counterparts. They are more starvation resistant.

This model suggests the following picture for which parameters provide the observed variation in fat and leptin in human populations (See supplemental information for mathematical analysis). When steady-state fat is higher than the critical fat level, the main parameter that controls fat level is leptin resistance $K_{L}$ (Figures 5C and 5D). When steady-state fat level is lower than the critical level, the main parameter that determines fat is the maximal satiety $u_{\max}$ (Figures 5E and 5F). Because the critical fat level rises with leptin resistance, individuals with severe leptin resistance can lie below the critical fat level (Methods).

**Figure 6:**
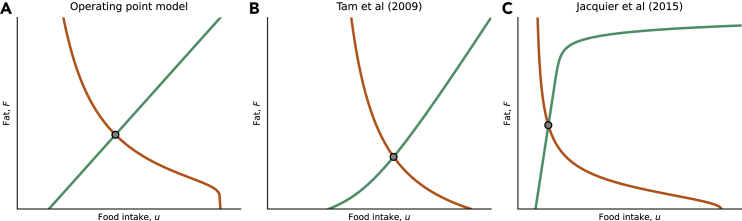
Diet and appetite can be derived from different leptin-based mathematical models (A) Operating point model presented in this paper. (B) Diet and appetite lines derived from the model of Tam et al. and (C) the model of Jacquier et al.

The leptin fat model of Equations 3, 4, 5, 6, 7, and 8 above can be made more realistic by adding additional considerations such as leptin entry to the brain, its effect on energy balance and other hormones, neuromodulators and neuronal circuits. We note however, that the present concepts of diet lines and appetite lines can help to understand more complex mathematical models. To demonstrate this, we analyzed two previous models^27^^,^^28^ (supplemental information). The model of Tam et al. without leptin resistance dynamics has 4 equations and 12 parameters, and the model of Jacquier et al., has 3 equations and 16 parameters. The models include features such as leptin transport to the brain, leptin regulation on energy expenditure and fat-free mass dynamics. We find that both models can be interpreted to produce diet and appetite lines, and thus fit the present conceptual rules, although the precise shapes of the lines depend on the model (Figure 6). Neither model can explain the quadratic relation between leptin fat in humans, because they lack the dependence of leptin production on recent food intake.

## Discussion

We presented a graphical model for food intake and fat dynamics which provides rules for interventions that explain a large set of rodent experiments. Interventions can be classified as affecting the diet line – the steady-state fat at a given food intake, or the appetite line – the *ad libitum* daily food intake at a given fat level. Interventions that affect the diet line change fat and food intake in opposite directions; those that affect the diet change fat and food intake in the same direction. The latter also show dynamical overshoots or undershoots in food intake over days. These rules result from general principles based on the fact that the diet line has increasing slope and the appetite line a decreasing slope. We also provide a minimal mechanistic model which defines the lines based on the leptin control system, explaining the quadratic dependence of leptin on fat in humans.

Opposing effects on food intake and fat are caused by interventions that shift the diet line. These interventions affect energy balance and hence affect the steady-state fat at a given food intake. A prime example is exercise: Exercising animals lose fat but increase food intake. Other examples include hyperthyroidism and changes in basal metabolic rate such as those caused by changes in temperature. A hallmark of hyperthyroidism in rodents and humans is weight loss despite increased appetite.^67^

Coherent effects on food intake and fat are caused by interventions that affect the appetite line. Such interventions do not affect energy balance directly but instead affect feeding behavior at a given fat level. These interventions include alterations of the leptin pathway, leptin resistance and appetite/satiety affecting nutrients such as deity fibers and increased food variety at constant caloric density. These interventions raise or lower both fat and food intake. Unlike diet line interventions, the appetite interventions also show an overshoot or undershoot in food intake that lasts for several days before approaching its new steady state.

These rules can help to diagnose more complex interventions. For example, estrogen supplementation in ovariectomized rats lowers fat but leaves steady state food intake nearly unchanged, after it adapts from an undershoot.^85^^,^^86^ This can be explained by a simultaneous change in both diet and appetite lines, in which estrogen reduces appetite but increases energy expenditure. These effects of estrogen on appetite and energy expenditure have been experimentally demonstrated.^87^^,^^88^^,^^89^

The rules also bear on changes during aging. Basal metabolic rate in humans drops with age,^90^^,^^91^ and thus shifts the diet line. This predicts that fat should rise and food intake should drop with age. This pattern matches observation on average.^92^^,^^93^ Exceptions to this pattern may point to additional perturbations of the diet and appetite lines which may occur in specific pathologies.

The present concept of the *operating point* can help integrate two conflicting concepts in fat regulation. These concepts are the set point and the settling point.^26^ A set point model typically assumes strong genetic and humoral control of body weight characterized by a feedback system designed to return weight to a constant “body inherent” weight. In contrast, a “settling point” model does not include a feedback controller but instead relies on the balance of food intake and energy expenditure to allow the fat level to reach a steady state that changes with conditions. The present operating point model includes aspects of both, a feedback regulator that pushes fat toward an operating point (the appetite line), and an energy balance that can shift the fat steady state according to food intake and energy expenditure (the diet line). This provides both control of weight around a nearly constant point over long periods, and the possibility for change of fat levels.

The present operating point model also relates to the concept of the dual intervention point,^94^^,^^95^^,^^96^ which suggests that there is not a single set point. Instead, there are upper and lower boundaries that define the weight/fat at which active physiological regulation becomes dominant, and between which there is only weak or no physiological regulation of weight. In the present operating point model, the appetite line shows high slopes at very high and very low fat levels. Food intake hits a ceiling at low fat and offers the strongest control to return fat to an intermediate level. This low-fat intervention point is reached at a fat level $F_{c}$ that is proportional to leptin resistance $K_{L}$. This makes a prediction that leptin resistant organisms reach the low intervention point and turn on anti-starvation defenses sooner (at a higher level of fat) than non-resistant organisms. Thus, it’s harder to diet and lose fat when severely leptin resistant. At high fat, food intake likewise drops rapidly to low levels, providing a strong restoring force to reduce fat to an intermediate level. These extreme controls can be argued to provide a rapid return to intermediate fat, a strategy that is optimal in certain settings known as bang-bang control.^97^^,^^98^^,^^99^

One may ask what measurements might be most relevant in the human case, given the problematic nature of monitoring food intake and the effects of social and psychological factors. The present approach suggests that measurements of leptin level together with fat level might provide a good diagnostic, based on a model that explains the quadratic dependence of leptin on fat in human populations. Such measurements might help to characterize the parameters for each individual, similar in spirit to the use of glucose and insulin blood tests together with minimal models to evaluate insulin resistance and beta cell function in diabetes.^100^ The present model points to variation in leptin resistance and maximal satiety level as primary causes of population difference in fat level. It may be used to potentially diagnose differences in leptin production and removal parameters, and parameters related to energy balance such as the conversion rate of food to fat.

The operating point model suggests experimental tests that can refute it. One experiment with perhaps non-intuitive predictions is as follows. Compare animals that reach the same steady-state fat under two different interventions, one that shifts the diet line (such as shift to cold temperature), and the other that shifts the appetite line (such as a leptin agonist). The former is predicted to show monotonic increase in food intake toward a final high intake level, and the latter is predicted to show an undershoot in food intake that settles to a lower final intake level, despite matched levels of initial and final body fat.

The operating point allows an adjustable set point for body weight allowing adaptation to times of food abundance or scarcity. An adjustable set point can also accumulate fuel stores to feed a growing fetus.^101^ Such homeostatic circuits with adjustable operating points are vulnerable to dysregulation because they are designed to be adjustable. In the modern environment, adjustable operating points may have contributed to the current obesity epidemic. Other examples are the adjustable sizes of endocrine glands, such as those in the HPA axis, which provide dynamic compensation for physiological changes, but can result in dysregulation.^102^^,^^103^^,^^104^^,^^105^^,^^106^

It would be important to extend the present model also to the control of fat-free mass (FFM). FFM is regulated by its own feedback circuitry, primarily by the protein content of food intake. One can envisage analogous diet and appetite lines for the case of FFM, which can be measured experimentally. It would be interesting to study the interaction of the fat and FFM control systems to gain a complete understanding of body weight regulation. For example, the work of Dulloo et al. points to the importance of FFM in hyperphagia after starvation, which may cause long term changes in the fat operating point.^20^^,^^53^^,^^70^^,^^107^^,^^108^

In summary the present rules and operating point model can help to make sense of complex interventions for body fat. They can potentially diagnose the causes for aberrant fat loss or gain by means of measurement of food intake or leptin levels, and help to design useful interventions.

### Limitations of the study

This study neglects, for the purpose of simplicity, known interactions in the physiology of fat mass control. These include the interactions of the regulatory system with fat-free mass, the effects of leptin on basal metabolic rate and specific mechanism of leptin resistance and their dependence on conditions. This study provides a model calibrated primarily on rodent data; its full calibration in humans would require additional studies.

## STAR★Methods

### Key resources table

**Table:** 

REAGENT or RESOURCE	SOURCE	IDENTIFIER
**Deposited data**		

	Harris et al.^29^	https://github.com/alonbar110/Rules-for-body-fat-interventions. https://doi.org/10.5281/zenodo.7515820
	Considine et al.^24^	https://github.com/alonbar110/Rules-for-body-fat-interventions. https://doi.org/10.5281/zenodo.7515820

**Software and algorithms**		

Python version 3.9.7	Python Software Foundation	https://www.python.org
Source code	This paper	https://github.com/alonbar110/Rules-for-body-fat-interventions. https://doi.org/10.5281/zenodo.7515820

### Resource availability

#### Lead contact

Further information and requests for resources should be directed to and will be fulfilled by the lead contact Uri Alon (uri.alon@weizmann.ac.il).

#### Materials availability

This study did not generate new unique reagents.

### Experimental model and subject details

This study is computational and does not included any experimental model or subjects.

### Method details

#### Database of rodent experiments

We compiled a database of well-controlled experiments on rodents performed in the past five decades, collected by a literature search and curated manually. We scanned the literature for papers that measured the effect of an intervention on both food intake and on fat or weight. We compiled the results of 90 interventions from 67 publications (Table S1). Food intake was measured in grams or calories per day. Exceptions included three experiments that treated rodents with either leptin, beta-glucan or Oxyntomodulin.^16^^,^^41^^,^^46^ They show reduction in both food intake and weight, but didn’t observe an undershoot. An experiment that treated rodents with MCH.^11^ It showed an increase in both food intake and weight, but overshoot was not observed. In addition, three experiments that followed the effect of an access to a running wheel found a surprising undershoot in food intake, although food intake was eventually increased.^4^^,^^9^^,^^109^

##### Analysis of Harris et al^29^data

Weight and food intake data-points were extracted from Harris et al., Figures 1 and 2.^29^ Fat mass data-points were extracted from Figure 4 (middle panel). Data-points were extracted using GRABIT via MATLAB.^110^ Next, we interpolated the data-points to provide a common timescale of 1 day. Because rats constantly gain weight as they grow, we normalized each experimental group by the control (Figures 2A and 2B). This also helped to correct for the aversive effect of tube-feeding that is also seen in the 100% control group. The controlled-feeding part of the experiment can be used to draw the diet line experimentally. The experiments provide 3 points on the phase portrait. Each point is the daily food intake relative to the control (40%, 100%, and 160%) and the resulting fat relative to control at the end of the controlled feeding. The recovery phase of the experiment enabled us to draw the appetite line. Each point is the relative fat and the relative daily *ad-libitum* feeding (Figure 2C).

##### The normalized phase portrait

The normalized phase portrait is a transform of the system’s phase portrait. In this space, food intake u and fat mass F are expressed in fold change relative to the control group.

To fit the points on the diet line, we used Equation 2, replacing the parameter groups $\frac{a_{F}}{\gamma_{F}},\frac{\gamma_{E}}{\gamma_{F}}$ with a,b respectivly:

(Equation 9) F=a·u−b

To fit the points on the appetite line we used Equation 7. We replaced the parameter group $\frac{K_{L}\gamma_{L}}{a_{L}}$ with c:

(Equation 10) $$F=c\cdot \frac{1}{u}\;{\left(\frac{u_{\max}}{u}-1\right)}^{\frac{1}{n}}$$

Fitting the diet (N=3 time points) (Equation 9) and appetite (N=39 time points) (Equation 10) lines to Harris et. al., provide estimation for the parameters of the normalized phase portrait. Due to the small number of points to fit the diet line, we could not estimate the error for a,b. Error is given by the standard deviation of the estimated value.

$$a=\frac{a_{F}}{\gamma_{F}}=1.56,b=\frac{\gamma_{E}}{\gamma_{F}}=0.49,c=\frac{K_{L}\gamma_{L}}{a_{L}}=1\pm 0.05$$

$$u_{\max}=1.38\pm 0.08$$

n=7.1±3.8

##### Model solutions

The solution of the model, namely the operating point, is given by the intersection of the diet and appetite line. At the limit of n→∞, we solve the model analytically in two regions of the appetite line:

First, from Equations 2, 8, and 5, for $F>F_{c}$ we find that:

(Equation 11a) $$u_{st}=\frac{\sqrt{4K_{L}\gamma_{F}\gamma_{L}a_{F}+\gamma_{E}^{2}a_{L}}}{2a_{F}\sqrt{a_{L}}}+\frac{\gamma_{E}}{2a_{F}}$$

(Equation 11b) $$F_{st}=\frac{\sqrt{4K_{L}\gamma_{F}\gamma_{L}a_{F}+\gamma_{E}^{2}a_{L}}}{2a_{F}\sqrt{a_{L}}}-\frac{\gamma_{E}}{2a_{F}}$$

(Equation 11c) $$L_{st}=K_{L}$$

So far we analyzed the model under the constraint ${F>F}_{c}$. Generally, in healthy individuals, $F_{c}$ is lower than $F_{st}$. However, when leptin resistance increases, the critical fat level $F_{c}$ (Equation 8) increases and the operating point shifts to the right along the diet line (Figures 2B and 5C). When leptin resistance crosses a critical value $K_{crit}$, $F_{c}$ becomes higher than $F_{st}$. The diet line intersects the vertical region of the appetite line. Any additional increase in $K_{L}$ will not change the operating point (Figure 5E). To find $K_{crit}$, we solve the equality were $F_{c}$ is equal to the diet line at $u=u_{\max}$ (Equations 2 and 8):

(Equation 12) $$K_{crit}=\frac{a_{L}u_{\max}}{\gamma_{L}\gamma_{F}}\left(a_{F}u_{\max}-\gamma_{E}\right)$$

Therefore, for $K_{L}\geq K_{crit}$, $F_{st}<F_{c}\text{,}$ the steady state of the system becomes:

(Equation 13a) $$u_{st}=u_{\max}$$

(Equation 13b) $$F_{st}=\frac{a_{F}}{\gamma_{F}}u_{\max}-\frac{\gamma_{E}}{\gamma_{F}}$$

(Equation 13c) $$L_{st}=\frac{a_{L}}{\gamma_{L}\gamma_{F}}u_{\max}\left({a_{F}u}_{\max}-\gamma_{E}\right)$$

##### Leptin-fat phase portrait

We break the feedback loop down to two arms described by two nullclines, which we call the leptin and fat lines. The leptin line is steady state leptin L at a given fat level F. This is the steady state leptin expression from Equation 5:

$$L=\frac{a_{L}}{\gamma_{L}a_{F}}F\left(\gamma_{F}F+\gamma_{E}\right)$$

The fat line is steady state fat if leptin is held constant (substituting Equation 6 into Equation 2):

(Equation 14) $$F=\frac{u_{\max}}{1+{\left(\frac{L}{K_{L}}\right)}^{n}}\frac{a_{F}}{\gamma_{F}}-\frac{\gamma_{E}}{\gamma_{F}}$$

We assume that n is very large, n→∞, giving a piecewise-linear expression for the fat line:

(Equation 15) $$F=\left\{ \begin{array}{c} \frac{a_{F}}{\gamma_{F}}u_{\max}-\frac{\gamma_{E}}{\gamma_{F}},L<K_{L}\\ -\frac{\gamma_{E}}{\gamma_{F}},L>K_{L} \end{array}\right.$$

We note that although this solution is mathematically valid, fat can’t reach negative value. Hence, the negative solution for fat represents a state of diminished fat stores and breakdown of lean mass to support body energetic needs.

#### Model sensitivity to the parameters, related to model solutions in the method section

At the limit n→∞, the operating point has two possible solutions depending on the regime of the appetite line where the lines intersect.

In first regim, $F_{st}>F_{c}$ (Equation 11), the only parameter that provides the variation in both fat and leptin, together with a quadratic relation is $K_{L}$. The lack of $u_{\max}$ from the operating point expression in this case means that the operating point is insensitive to variation in this parameter. Variation in the other model parameters, results in new quadratic association between fat mass and leptin and explains the variation in the leptin-fat data that is orthogonal to the quadratic relation.

In the second solution, $F_{st}<F_{c}$ ((Equation 13a), (Equation 13b), (Equation 13c)), we find a sharp transition in the model sensitivity to the parameters. In this case the only parameter that provides the variation in both fat and leptin, together with a quadratic relation, is $u_{\max}$, wherehas $K_{L}$ has no effect on this relation. Variation in other model parameters, results in new quadratic association between fat mass and leptin variation in the other model parameters, results in new quadratic association between fat mass and leptin and explains the variation in the leptin-fat data that is orthogonal to the quadratic relation.

So far we used the approximation of n→∞. This enable us to deal with a simple mathematical form for the lines, with appetite line that is defined in two regions. To complete this analysis, we also numerically computed the model’s sensitivity to parameters as function of n. To do so, we calculated the partial derivative of the operating point with respect to each parameter as function of:

At the operating point, all variables are in steady state. Solving the diet line (Equation 2) as function of F, and substituting food intake equation (Equation 6) and leptin steady state (Equation 5) provide the following equation for $F_{st}$:

(Equation 16) $$0=F\left(K_{L}^{n}\gamma_{F}\gamma_{L}^{n}a_{F}^{n}+\gamma_{F}{\left(F^{2}\gamma_{F}a_{L}+F\gamma_{E}a_{L}\right)}^{n}\right)+K_{L}^{n}\gamma_{L}^{n}\gamma_{E}a_{F}^{n}-K_{L}^{n}\gamma_{L}^{n}a_{F}a_{F}^{n}u_{\max}+\gamma_{E}{\left(F^{2}\gamma_{F}a_{L}+F\gamma_{E}a_{L}\right)}^{n}$$

Next, we consider $F_{st}$ as function of X, where X can be any parameter, and calculate the partial derivative of the characteristic equation with respect of X, $\frac{\partial F\left(X\right)}{\partial X}$. For example, for $\alpha_{F}$ we find:

(Equation 17) $$\frac{\partial F\left(\alpha_{F}\right)}{\partial \alpha_{F}}=-\frac{a_{F}^{n-1}{\left(K_{L}\gamma_{L}\right)}^{n}\left(\gamma_{F}nF\left(a_{F}\right)+\gamma_{E}n-a_{F}nu_{\max}-a_{F}u_{\max}\right)F\left(a_{F}\right)}{K_{L}^{n}\gamma_{F}\gamma_{L}^{n}a_{F}^{n}F\left(a_{F}\right)+2\gamma_{F}n{\left(a_{L}\left(\gamma_{F}F\left(a_{F}\right)+\gamma_{E}\right)F\left(a_{F}\right)\right)}^{n}F\left(a_{F}\right)+\gamma_{F}{\left(a_{L}\left(\gamma_{F}F\left(a_{F}\right)+\gamma_{E}\right)F\left(a_{F}\right)\right)}^{n}F\left(a_{F}\right)+\gamma_{\epsilon }n{\left(a_{L}\left(\gamma_{F}F\left(a_{F}\right)+\gamma_{E}\right)F\left(a_{F}\right)\right)}^{n}}$$

Using a fixed parameter set, we numerically solved the operating point for range of values for n. Thus, for every value of n, we find a numerical value $F_{st}\left(n\right)$. Then, for each value of n, we numerically calculate the partial derivative, substituting F(X) with $F_{st}\left(n\right)$.

Eventually, in order to have meaningful units we divide this value by $F_{st}\left(n\right)$. Thus, we represent the partial derivative in proportion to the steady state. This is the log-partial derivative: $\frac{\partial \mathrm{Log}\left(F\left(X\right)\right)}{\partial \mathrm{Log}\left(X\right)}=\frac{X}{F\left(X\right)}\frac{\partial F\left(X\right)}{\partial X}$ .

We repeat this process for L and u, using their respective equations:

(Equation 18) $$0=K_{L}^{2n}\gamma_{E}a_{L}u_{\max}-K_{L}^{2n}a_{F}a_{L}u_{\max}^{2}+K_{L}^{n}L^{n}\gamma_{E}a_{L}u_{\max}+L\left(K_{L}^{2n}\gamma_{F}\gamma_{L}+2K_{L}^{n}L^{n}\gamma_{F}\gamma_{L}+L^{2n}\gamma_{F}\gamma_{L}\right)$$

(Equation 19) $$0=-K_{L}^{n}\gamma_{F}^{n}\gamma_{L}^{n}u_{\max}+u\left(K_{L}^{n}\gamma_{F}^{n}\gamma_{L}^{n}+{\left(-\gamma_{E}a_{L}u+a_{F}a_{L}u^{2}\right)}^{n}\right)$$

We used two parameter sets to represent two distinct regions. For the first region, we used the baseline parameter set. In this set, $F_{st}$ is larger than $F_{c}$ at the approximation of n→∞. For the second region we calculate $K_{crit}$ from the baseline set, and replaced $K_{L}$ value with $2\times K_{crit}$. Hence, this set represent the region where $F_{c}$ is significantly larger than $F_{st}$.

This analysis provides the relative sensitivity of $F_{st},L_{st}$ and $u_{st}$ for each parameter as a function of n in two distinct regions of the parameter space (Figure S1).

##### Modeling

To model the shape and the dynamics of the phase portrait (Figures 1,3, 4, and 5) we used the simplified version of the operating point model, assuming that n→∞. In this case, the exact expression for the diet line, appetite line, critical fat level and the operation point and is given by Equations 2, 8, and 11. The expression for the fat and leptin lines is given by Equations 5 and 15. We used a default set of parameters for most of the figures. Parameter values are available in Table 4.

In Figure 3, we increased $\gamma_{E}$ to reflect an increase in energy expenditure due to exercise (Figure 3A) and $K_{L}$ to reflect increase in leptin resistance (Figure 3B).

In Figure 4, to model the system’s dynamics of food intake and fat change, we simulated the operating point model equations for 50 days: In Figures 4A and 4B we selected initial conditions that represent a period of over/under-feeding, this is given by multiplying the steady state food intake (Equation 11) by 1.4 or 0.7, and solving fat using the diet line (Equation 2):

$$\left(F_{overfeeding}=\frac{a_{F}}{\gamma_{F}}1.4u_{st}-\frac{\gamma_{E}}{\gamma_{F}},u_{overfeeding}=1.4u_{st}\right)$$

$$\left(F_{underfeeding}=\frac{a_{F}}{\gamma_{F}}0.7u_{st}-\frac{\gamma_{E}}{\gamma_{F}},u_{underfeeding}=0.7u_{st}\right)$$

In the remaining panels, we again increased $K_{L}$ to model increase in leptin resistance (Figure 4C) and $\alpha_{F}$ to model a shift to a high fat diet (Figure 4D). In these cases, the initial condition is the operating point of the default parameters set. To simulate the dynamics, we used the model's equations (Equations 1, 3, and 6) with n=100. Simulations were done in Python using the scipy odeint solver.

In Figures 5C and 5E, we used a range of $K_{L}$ values and plotted the appetite and diet lines, the operating point and fat critical level $F_{c}$. In Figure 5D, we plot the fat and leptin lines, varying $K_{L}$ within a range in which satisfies $K_{L}\leq K_{crit}$. In Figure 5F, we calculated $K_{crit}$ of the default parameter set, and plotted the fat and leptin lines with $K_{L}=2K_{crit}$. Then, we varied $u_{\max}$ within a range in which satisfies $K_{L}\geq K_{crit}$.

In Figure 6A, we model the lines and operating point using n=6 and the default parameter set.

#### Reanalysis of Considine et al.^24^

Fat and leptin data-points were extracted from Considine et al., Figure 1^24^ using GRABIT via MATLAB.^110^ Next we fit the leptin line, a quadratic curve, to the data points (N=70) (Equation 5):

(Equation 20) $$L=aF^{2}+bF,a=\frac{a_{L}\gamma_{F}}{\gamma_{L}a_{F}},b=\frac{a_{L}\gamma_{E}}{\gamma_{L}a_{F}}$$

We find that a=0.016±0.003,b=0.062±0.143. (Mean ± SD)

We compare this result to a linear fit, L=cF, using Akaike information criteria (AIC) and Pearson correlation. The quadratic model had lower AIC score and higher Pearson correlation ($AIC=1143,r^{2}=0.72)$ comparing to the linear model ($AIC=1163,r^{2}=0.68)$. This indicate that the data-points support quadratic relation rather linear.

#### Reanalysis of previous mathematical models, related to Figure 6

We analyzed two previous models. These models are more complex than the operating point models. They include features such as leptin transport to the brain, leptin regulation on energy expenditure and fat-free mass dynamics. Note: to maintain universality we decided to annotate food intake as u, fat mass as F and leptin as L regardless of their original notation. Otherwise we kept each model’s original notation.

#### Tam et al., 2009

Tam et al., developed a system of ordinary differential equations to describe the effects of leptin on various aspects of energy metabolism. They carried out and compared simulations for two separate systems - with and without control by an explicit set-point. We reanalyzed Tam’s model without control by an explicit set-point. This model is given by a set of 4 equations (Equations 22, 23, 24, and 27):

Body weight is the sum of fat mass F and fat-free mass FFM. This equation assumes FFM is relatively constant. $\rho_{F}$ is the energy density of fat.

(Equation 21) $$BM=F+FFM=\frac{E}{\rho_{F}}+FFM$$

Leptin is produced by fat cells and removed by the kidneys:

(Equation 22a) $$\frac{dL_{plasma}}{dt}=\frac{F{\times R}_{syn}-GFR\times RenClearance\times L_{plasma}}{BloodVolume}$$

We simplify this expression:

$$\frac{R_{syn}}{BloodVolume}=\alpha_{L},\frac{GFR\times RenClearance}{BloodVolume}=\gamma_{L}$$

(Equation 22b) $$\frac{dL_{plasma}}{dt}\alpha_{L}F-{\gamma_{L}L}_{plasma}$$

In addition, Tam et al., model brain leptin levels. Plasma leptin enters the brain both by saturable receptors and by linear diffusion:

(Equation 23) $$L_{brain}=\frac{L_{plasma}k_{1}}{L_{plasma}+k_{2}}+L_{plasma}k_{3}$$

Brain leptin reduce food intake u with Michaelis-Menten equation to represent this relationship:

(Equation 24) $$u=k_{4}\left(1-\frac{L_{brain}}{L_{brain}+k_{5}}\right)$$

Energy intake is proportional to u by its metabolizable energy content $\rho_{food}$

(Equation 25) $$E_{in}=\rho_{food}u$$

Energy expenditure is proportional to total body weight, with additional effect of leptin

(Equation 26) $$E_{out}=BMk_{6}\left(1+\frac{L_{brain}k_{7}}{L_{brain}+k_{8}}\right)$$

Overall energy balance is

(Equation 27) $$\frac{dE}{dt}=E_{in}-E_{out}=\rho_{food}u-BMk_{6}\left(1+\frac{L_{brain}k_{7}}{L_{brain}+k_{8}}\right)$$

We analyze these equations and derive the diet and appetite lines:

To calculate the diet line, like in the operating point model, we fix food intake and we wait until steady-state $\frac{dE}{dt}=0$. Solving (Equation 23) for food intake u yields the following expression:

(Equation 28) $$u=\frac{BMk_{6}\left(L_{brain}k_{7}+L_{brain}+k_{8}\right)}{\rho_{food}\left(L_{brain}+k_{8}\right)}$$

We assume a quasi-steady state for plasma leptin. From (Equation 22b) we find that $L_{plasma_{qst}}=\frac{a_{L}F}{\gamma_{L}}$. Plugin $L_{plasma_{qst}}$ into (23):

(Equation 29) $$L_{brain}=\frac{Fa_{L}\left(\gamma_{L}k_{1}+k_{3}\left(Fa_{L}+\gamma_{L}k_{2}\right)\right)}{\gamma_{L}\left(Fa_{L}+\gamma_{L}k_{2}\right)}$$

Thus, the explicit expression for Tam’s diet line is:

(Equation 30) $$u=\frac{k_{6}\left(F+FFM\right)\left(Fa_{L}k_{7}\left(\gamma_{L}k_{1}+k_{3}\left(Fa_{L}+\gamma_{L}k_{2}\right)\right)+Fa_{L}\left(\gamma_{L}k_{1}+k_{3}\left(Fa_{L}+\gamma_{L}k_{2}\right)\right)+\gamma_{L}k_{8}\left(Fa_{L}+\gamma_{L}k_{2}\right)\right)}{\rho_{food}\left(Fa_{L}\left(\gamma_{L}k_{1}+k_{3}\left(Fa_{L}+\gamma_{L}k_{2}\right)\right)+\gamma_{L}k_{8}\left(Fa_{L}+\gamma_{L}k_{2}\right)\right)}$$

Unlike the operating point model, this is not a linear line. However, it approximates a linear line at very low and high fat levels:

At very low fat mass, $1\gg \frac{L_{brain}k_{7}}{L_{brain}+k_{8}}$ and the diet line becomes:

(Equation 31) $$u=\frac{k_{6}\left(F+FFM\right)}{\rho_{food}}$$

At very high fat mass, $L_{brain}\gg k_{8}$ the diet line becomes:

(Equation 32) $$u=\frac{k_{6}\left(F+FFM\right)}{\rho_{food}}\left(1+k_{7}\right)$$

The appetite line, like in the operating point model, is food intake at given fat. Plugin (Equation 29) into (Equation 24), we get:

(Equation 33) $$u=\frac{\gamma_{L}k_{4}k_{5}\left(Fa_{L}+\gamma_{L}k_{2}\right)}{Fa_{L}\left(\gamma_{L}k_{1}+k_{3}\left(Fa_{L}+\gamma_{L}k_{2}\right)\right)+\gamma_{L}k_{5}\left(Fa_{L}+\gamma_{L}k_{2}\right)}$$

To plot the lines on the phase portrait (Figure 5C), we used the parameters from the original paper (Tam et al, 2009^28^ - Table 1)

#### Jacquier et al., 2015

The purpose of this work was to propose a theoretical model of leptin resistance development, and to qualitatively study the dynamics behind the development of leptin resistance and its influence on food intake and body weight. This model focuses on fat mass, fat-free mass and food intake, and takes into account plasma leptin and leptin receptor dynamics to mediate food intake. We reanalyzed Jacquier’s model with constant receptor density. This model is given by a set of 3 equations (Equations 38, 39, and 42):

Variations of FM and FFM are correlated with the difference between energy intake (EI) and energy expenditure (EE). The parameters $\rho_{FFM}$ and $\rho_{FM}$ denote the caloric densities of fat-free mass and fat mass respectively.

(Equation 34) $$\frac{dF}{dt}=\frac{EI-EE}{\rho_{FFM}\Omega +\rho_{F}}$$

The function Ω denotes the body composition function, describing the relationship between fat mass and fat-free mass.

(Equation 35) $$\Omega =\gamma_{\Omega }\left(1+\alpha e^{\kappa F}\right)$$

Energy intake corresponds to the caloric content of food intake u characterized by the caloric density $\gamma_{E}$ of the food. Energy expenditure is assumed to be proportional to fat mass and fat-free mass with a basal energy expenditure ξ and a rate of energy expenditure η. The energy balance is then defined as:

(Equation 36) $$EI-EE=\gamma_{E}u-\eta \left(\rho_{FFM}FFM+\rho_{F}F+\xi \right)$$

FFM is explicitly obtained from F and initial conditions in the original paper. Where C is a function of the initial conditions.

(Equation 37) $$FFM=\gamma_{\Omega }\left(\kappa F+\alpha e^{\kappa F}\right)\kappa +C$$

Together, we get a differential equation for fat mass F:

(Equation 38) $$\frac{dF}{dt}=\frac{\gamma_{E}u-\eta \left(\left(\rho_{F}+\rho_{FFM}\gamma_{\Omega }\right)F+\frac{\rho FFM\alpha e^{\kappa F}}{\kappa }+\rho_{FFM}C+\xi \right)}{\rho_{FFM}\gamma_{\Omega }\left(1+\alpha e^{\kappa F}\right)+\rho_{F}}$$

Plasma leptin L is produced by adipocytes proportionally to fat mass

(Equation 39) $$\frac{dL}{dt}=\gamma_{L}F-\delta_{L}L$$

Jacquier et al., also provided an equation for the density of leptin receptors R in the hypothalamus. They assume that both production and degradation of R are increased by leptin. However, in this comparison, we choose to omit this interaction. We will regard R as a fixed parameter.

Activation of leptin receptors in the hypothalamus leads to a path-way controlling food intake. The response $\Phi_{R}\left(L\right)$ of the activation of leptin receptors can be described by a Hill function with $\varphi_{R}$ maximal response. (Note that we grouped the parameters φ and R)

(Equation 40) $$\Phi_{R}\left(L\right)=\varphi_{R}\frac{L^{n}}{L^{n}+\theta^{n}}$$

Jacquier et al., assume that food intake u is inhibited by the activation of leptin receptors

(Equation 41) $$\frac{du}{dt}=\frac{\gamma_{u}}{1+\Phi_{R}\left(L\right)}-\delta_{u}u$$

Together:

(Equation 42) $$\frac{du}{dt}=\frac{\gamma_{u}\left(L^{n}+\theta^{n}\right)}{L^{n}\left(1+\varphi_{R}\right)+\theta^{n}}-\delta_{u}u$$

Again, we analyze these equations and derive the diet and appetite lines:

We assume quasi-steady state for plasma leptin, from Equation 39 we get:

(Equation 43) $$L_{qst}=\frac{\gamma_{L}}{\delta_{L}}F$$

To calculate the diet line, like in the operating point model, we fix food intake and we wait until steady-state $\frac{dF}{dt}=0$. Solving (Equation 38) for u yields the following expression:

(Equation 44) $$u=\frac{\eta \left(\gamma_{\Omega }\rho_{FFM}\alpha e^{\kappa F}+\kappa \left(C\rho_{FFM}+F\gamma_{\Omega }\rho_{FFM}+F\rho_{F}+\xi \right)\right)}{\gamma_{E}\kappa }$$

The appetite line is food intake at given fat. Plugin (Equation 43) into (Equation 42) and solving for u we find:

(Equation 45) $$u=\frac{\gamma_{u}\left(F^{n}\gamma_{L}^{n}+\delta_{L}^{n}\theta^{n}\right)}{\delta_{u}\left(F^{n}\gamma_{L}^{n}\varphi_{R}+F^{n}\gamma_{L}^{n}+\delta_{L}^{n}\theta^{n}\right)}$$

To plot the lines on the phase portrait (Figure 5B), we used the parameters from the original paper (Jacqueir et al, 2015^27^ - Table 3)

### Quantification and statistical analysis

All statistical tests were performed in python and are similarly described in the figure captions. Statistical details of the analysis are found in the STAR Methods section.

## Data Availability

• The source code used to perform the analysis is available at the GitHub repository as of the date of publication. Github repository: https://github.com/alonbar110/Rules-for-body-fat-interventions. DOI is listed in the key resources table. • The data used to perform the analysis is available at the GitHub repository as of the date of publication. Github repository: https://github.com/alonbar110/Rules-for-body-fat-interventions. DOI is listed in the key resources table.

## Supplemental information

Document S1. Figure S1

Table S1. Database of rodent experiments, related to STAR Methods
