# Supplementary material for: Rules for body fat interventions based on an operating point mechanism
Source: iScience. 2023 Jan 25;26(2):106047. doi: 10.1016/j.isci.2023.106047 (PMC9929596; doi:10.1016/j.isci.2023.106047)
Supplement: Document S1. Figure S1 [file mmc1.pdf]

iScience, Volume *26*

Supplemental information

Rules for body fat interventions based on an operating point mechanism

Alon Bar, Omer Karin, Avi Mayo, Danny Ben-Zvi, and Uri Alon

Operating point sensitivity for each parameter as a function of n, Related to STAR Methods.

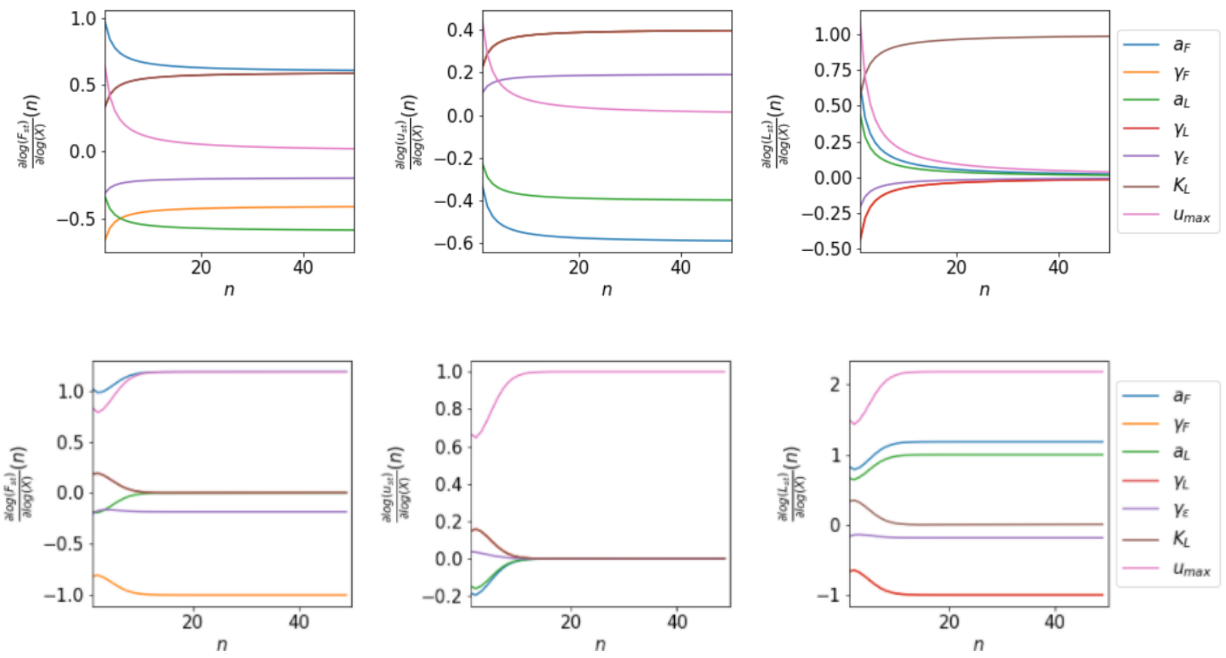

Figure S1. Operating point sensitivity for each parameter as a function of n. (A) Operating point with $F_{st}>F_c$. (B) Operating point with severe leptin resistance, such that $F_c>F_{st}$.
